# Abnormal Intrinsic Functional Interactions Within Pain Network in Cervical Discogenic Pain

**DOI:** 10.3389/fnins.2021.671280

**Published:** 2021-04-14

**Authors:** Hong Zhang, Dongqin Xia, Xiaoping Wu, Run Liu, Hongsheng Liu, Xiangchun Yang, Xiaohui Yin, Song Chen, Mingyue Ma

**Affiliations:** ^1^Department of Radiology, The Affiliated Xi’an Central Hospital, Xi’an Jiaotong University, Xi’an, China; ^2^Department of Ultrasound, The Affiliated Xi’an Central Hospital, Xi’an Jiaotong University, Xi’an, China; ^3^Department of Radiology, The Affiliated Xi’an XD Group Hospital, Shaanxi University of Traditional Chinese Medicine, Xi’an, China

**Keywords:** cervical discogenic pain, pain network, resting-state, functional connectivity, fMRI

## Abstract

Cervical discogenic pain (CDP) is mainly induced by cervical disc degeneration. However, how CDP modulates the functional interactions within the pain network remains unclear. In the current study, we studied the changed resting-state functional connectivities of pain network with 40 CDP patients and 40 age-, gender-matched healthy controls. We first defined the pain network with the seeds of the posterior insula (PI). Then, whole brain and seed-to-target functional connectivity analyses were performed to identify the differences in functional connectivity between CDP and healthy controls. Finally, correlation analyses were applied to reveal the associations between functional connectivities and clinical measures. Whole-brain functional connectivity analyses of PI identified increased functional connectivity between PI and thalamus (THA) and decreased functional connectivity between PI and middle cingulate cortex (MCC) in CDP patients. Functional connectivity analyses within the pain network further revealed increased functional connectivities between bilateral PI and bilateral THA, and decreased functional connectivities between left PI and MCC, between left postcentral gyrus (PoCG) and MCC in CDP patients. Moreover, we found that the functional connectivities between right PI and left THA, between left PoCG and MCC were negatively and positively correlated with the visual analog scale, respectively. Our findings provide direct evidence of how CDP modulates the pain network, which may facilitate understanding of the neural basis of CDP.

## Introduction

Cervical discogenic pain (CDP) is a chronic pain with clinical manifestations of pain in the head, neck, shoulder, and upper limbs, as well as pain associated with numbness. CDP is a common source of neck pain with a reported prevalence between 16% and 41% (Peng and DePalma, 2018). Cervical discs have an amount of nerve fibers that are prone to structural disruption and inflammatory reactions that make them susceptible to pain. CDP seriously affects physical and mental health as well as the life quality of patients (Tracy and Bartleson, 2010; Thoomes et al., 2012). Long-term CDP leads to functional abnormalities in sensorimotor processing, emotion, cognition, and memory (Montero-Homs, 2009; Linton, 2013; Denkinger et al., 2014). Despite such high prevalence, the neuropathology of CDP is still unclear.

The term “pain matrix” which is constituted of a set of brain areas mainly including the insula, cingulate cortex, parietal opercular and so on, is thought to be primarily involved in nociceptive processing (Isnard et al., 2011; Mouraux et al., 2011; Favilla et al., 2014). The insular cortex serves as an interface between internal and external stimuli for integrating multimodal information with rich connections to cortical and subcortical brain areas, and thus it is considered to be a core brain area of the pain network (Craig et al., 2000; Craig, 2002, 2003; Deen et al., 2011; Favilla et al., 2014). The posterior insula (PI) is a part of the lateral pain matrix and is considered a key area for pain perception and generation (Craig et al., 2000; Tracey, 2005; Favilla et al., 2014). Therefore, uncovering changes in functional interactions within the pain network may provide new insights into the neuropathology of CDP.

Resting-state fMRI is a non-invasive method of studying brain functional activities with blood oxygen level-dependent (BOLD) signals (Biswal et al., 1995; Fox and Raichle, 2007). Resting-state fMRI has been widely used to identify the intrinsic functional topography and networks of the brain (Cohen et al., 2008; Yeo et al., 2011; Wang et al., 2015, 2017, 2019a; Gao et al., 2020). Moreover, compared to task-fMRI, resting-state fMRI is an easier approach for detecting the intrinsic functional organization of the brain without complex task design and individual differences in finishing the task. Thus, resting-state has been widely applied to study the intrinsic functional abnormalities and treatment mechanisms for depression (Chen et al., 1997; Wu H. et al., 2016; Drysdale et al., 2017; Wang et al., 2018, 2019b,c, 2020), Alzheimer’s disease (Greicius et al., 2004; Liu et al., 2014; Wu Y. et al., 2016; Zhan et al., 2016), schizophrenia (Anticevic et al., 2014; Du et al., 2017; Fu et al., 2018), and so on.

In this study, 40 CDP patients and 40 age- and gender-matched healthy controls (HC) were used to identify the abnormalities of functional interactions within the pain network to reveal its neuropathology. First, the center coordinates of bilateral PI were used to define the pain network. Next, the pair-wise functional connections within the pain network were calculated and compared between CDP and HC to identify changed functional connectivities. Finally, correlation analyses were performed to identify the relationships between functional connections and clinical characteristics.

## Materials and Methods

### Participants

The current study included 40 right-handed CDP patients (F/M = 22/18; mean age = 53.6 ± 6.9 years) and 40 age- and gender-matched healthy controls (F/M = 22/18; mean age = 52.8 ± 7.6 years). CDP patients were diagnosed by one experienced orthopedist using physical and imaging examination. For CDP patients, the lesioned disc showed obvious reduced weighted T2 signals compared to the neighboring normal disc. Furthermore, only the pain caused by intervertebral disc disorder, not by segmental nerve disorder or radiation pain was considered to be CDP. The patients with disc degeneration and stenosis, cervical disc herniation, cervical spondylosis, hypertension, mental illness, diabetes, intracranial infection, craniocerebral trauma, MRI contraindication, and a history of chronic pain were excluded. 40 right-handed healthy controls without any hypertension, mental illness, intracranial infection, diabetes, craniocerebral trauma, and a history of chronic pain were included. The pain intensity was assessed with the visual analog scale (VAS) test, cognitive functions were assessed with the Montreal cognitive assessment scale (MoCA), and emotional states were assessed using the Hamilton depression scale (HAMD) and Hamilton anxiety scale (HAMA). Written informed consent was obtained from all the subjects, and this study was approved by the ethics committee of the affiliated Xi’an central hospital of Xi’an Jiaotong University and was in accordance with the Helsinki declaration and its later amendments or comparable ethical standards.

### Resting-State fMRI Data Acquisition

The resting-state fMRI data were acquired with a Philips 3.0T MRI scanner. All the subjects were asked to keep their eyes closed, not to move, and fall asleep. Foam pads and headphones were used to reduce head movement and scanner noise. The resting-state fMRI data was scanned using echo plane imaging (EPI) with the following parameters: repetition time (TR) = 2000 ms, echo time (TE) = 30 ms, flip angle = 90°, field of view = 230 × 230, acquisition matrix size = 64 × 64, 38 slices with 0.6 mm gap, the voxel size = 3.6 mm × 3.6 mm × 3.6 mm, 240 volumes.

### Resting-State fMRI Data Preprocessing

The resting-state fMRI data were preprocessed with the following steps: (1) the first 10 volumes were discarded to facilitate magnetization equilibrium effects; (2) the remaining images were realigned to the first volume to correct head motion; (3) all the images were normalized to standard EPI template and resampled to 3 mm^3^ isotropic voxels; (4) all the data were smoothed with 6 mm Gaussian kernel; (5) Friston 24-parameter of head motion, white matter, cerebrospinal fluid, and whole brain mean signals were regressed out; (6) the fMRI images were filtered by the frequency band of 0.01–0.08 Hz. To exclude head motion effects, a scrubbing approach was used to cut the bad images before 2 time points and after 1 time point exceeding the pre-set criteria of frame displacement (FD) > 0.5 (Power et al., 2012). All the subjects’ information, resting-state fMRI data acquisition and preprocessing can be found in our previous study (Ma et al., 2020).

### Definition of the Posterior Insula

**FIGURE 1:**
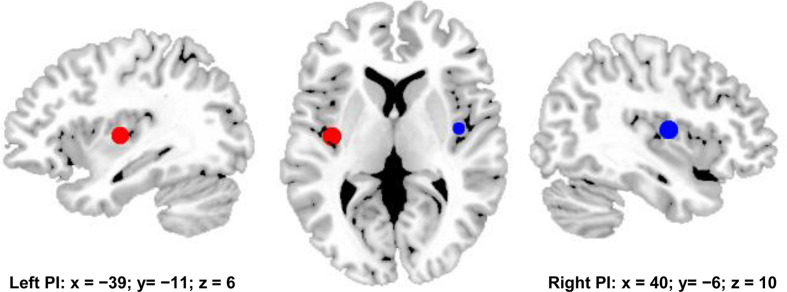
Bilateral posterior insula (PI) were used to define pain matrix. The center MNI coordinates were used to draw spheres with 6 mm radius for functional connectivity analyses. The center MNI coordinate for left PI is [–39, –11, 6], and the center MNI coordinates for right PI is [40, –6, 10].

The posterior insula (PI) is considered to be a core brain area of the pain network (Apkarian et al., 2005; Isnard et al., 2011). To define the pain network, whole brain functional connectivity analyses of bilateral PIs were employed to identify the functionally connected brain areas with PI. The PI seed areas were defined with the MNI center coordinates by drawing spheres with 6mm radius for whole brain resting-state functional connectivity analyses. The MNI center coordinate for left PI is [−39 −11 6], and the MNI center coordinate for right PI is [40 −6 10] (Figure 1) (Favilla et al., 2014).

### Seed-Based Functional Connectivity Analysis

First, seed-based functional connectivity analyses of bilateral PIs were performed to identify functional connectivity differences between CDP patients and healthy controls. Resting-state functional connectivity strength was measured using Pearson’s correlation coefficients. After whole brain functional connectivity maps were obtained, Fisher z transformations were used to transform functional connectivity maps to Z-maps to improve normality. Finally, two-sample t-tests with MoCA, HAMD, and HAMA as covariates were performed to identify the functional connectivity differences with bilateral PIs between CDP and healthy controls. The significant level was determined using a Gaussian random field (GRF) correction method with *p* < 0.05.

### Determination of Pain Network

To define the pain network, whole brain resting-state functional connectivity analyses of bilateral PIs were performed in healthy controls. After obtaining the whole brain functional connectivity maps for left and right PIs, one-sample *t*-tests and family wise error (FWE) correction with *p* < 0.05 and cluster size ≥ 100 were used to identify the brain regions which functionally connected to bilateral PIs. Finally, the peak MNI coordinates of these brain areas were obtained and used to draw 6 mm radius spheres for seed to target functional connectivity analyses.

### Functional Connectivity Analyses of Pain Network

After obtaining the pain network, seed to targets resting-state functional connectivity between each pair of brain regions belonging to the pain network were calculated. A Fisher z transformation was used to change the correlation coefficient to z value to improve normality. To identify the significant changes of functional connectivities within the pain network in CDP patients, two-sample t-tests were performed and the significance level was set at *p* < 0.05 with Bonferroni correction.

### Correlation Analyses

To explore the relationship between functional connectivities and clinical measurements, correlation analyses were performed between functional connectivities showing differences between CDP and healthy controls and VAS, MoCA, HAMD, HAMA, and duration of disease in CDP patients. The significance was set at *p* < 0.05 with Bonferroni correction.

## Results

### Clinical Characteristics

The well matched age (*p* = 0.78) and gender (*p* = 1) between CDP patients and healthy controls were shown in Table 1. Significantly decreased MoCA (*p* < 0.001) and significantly increased HAMD (*p* < 0.001), HAMA (*p* < 0.001) scores in CDP patients compared to healthy controls were found (Table 1).

**TABLE 1:** Demographic characteristics of cervical discogenic pain (CDP) and healthy controls (HC).

Characteristic	CDP (40, 22F/18M)	HC (40, 22F/18M)	*p* value
Age (Mean ± SD years)	53.6 ± 6.9	52.8 ± 7.6	0.78
VAS	6.73 ± 1.65		NA
MoCA	17.03 ± 1.83	26.38 ± 3.67	<0.001
HAMD	4.94 ± 3.95	0.91 ± 0.73	<0.001
HAMA	5.27 ± 3.78	0.77 ± 0.59	<0.001
Duration of pain (years)	3.25 ± 1.46		NA

*F, female; M, male; VAS, visual analog scale; MoCA, Montreal Cognitive Assessment; HAMD, Hamilton Depression Rating Scale; HAMA, Hamilton Anxiety Scale; NS, not significant; NA, not available.*

### Changed Whole Brain Functional Connectivity of Bilateral PIs

**FIGURE 2:**
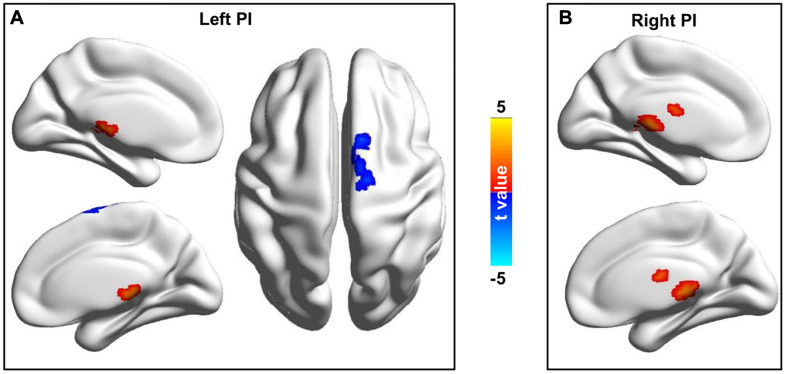
Changed resting-state functional connectivities of the bilateral posterior insula (PI) in CDP patients. **(A)** Left PI showed increased functional connectivities with the thalamus and decreased functional connectivities with the middle cingulate cortex/supplementary motor area in CDP patients compared to healthy controls. **(B)** Right PI showed increased functional connectivities with thalamus in CDP patients compared to healthy controls.

Whole brain functional connectivity analyses of bilateral PIs found bilateral PIs had significantly increased functional connectivities with bilateral thalamus (THA) and left PI additionally had decreased functional connectivity with the middle cingulate cortex (MCC)/supplementary motor area (Figure 2).

### Determining the Pain Network

**FIGURE 3:**
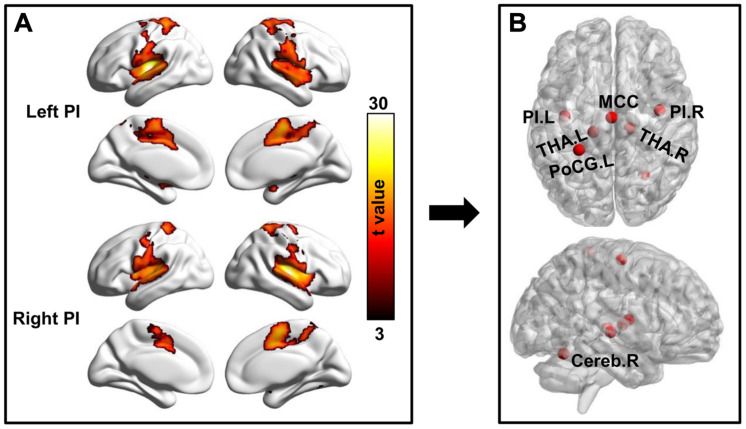
Definition of pain network. **(A)** Whole brain resting-state functional connectivity analyses of the bilateral posterior insula (PI) were performed and corrected using family wise error (FWE) correction method with *p* < 0.05 to identify the brain regions functionally connected to bilateral PIs. **(B)** Several brain areas including bilateral PI, bilateral thalamus (THA.L, THA.R), middle cingulate cortex (MCC), left postcentral gyrus (PoCG.L), and right cerebellum (Cereb.R) were identified and defined as pain network.

With bilateral PIs as seed regions, whole brain functional connectivity analyses in healthy controls identified five other brain regions showing significant functional connectivities. The five brain regions are MCC, left and right THA, left postcentral gyrus (PoCG), and right cerebellum (Cereb). In total, seven different regions consisted of the pain network and were used for seed to target functional connectivity analyses (Figure 3 and Table 2).

**TABLE 2:** The MNI peak coordinates of the pain network related brain regions, obtained with whole brain functional connectivity analyses of the bilateral posterior insula in healthy controls.

Brain regions	L/R	Abbreviation	Peak MNI coordinates		
			X	Y	Z
Posterior insula	L	PI	−39	−11	6
Posterior insula	R	PI	40	−6	10
Thalamus	L	THA	−15	−24	3
Thalamus	R	THA	15	−21	0
Postcentral gyrus	L	PoCG	−27	−39	66
Middle cingulate cortex	L/R	MCC	0	−12	60
Cerebellum	R	Cereb	27	−60	−18

*MNI, Montreal Neurological Institute; L, left hemisphere; R, right hemisphere.*

### Changed Functional Connectivities Within Pain Network

**FIGURE 4:**
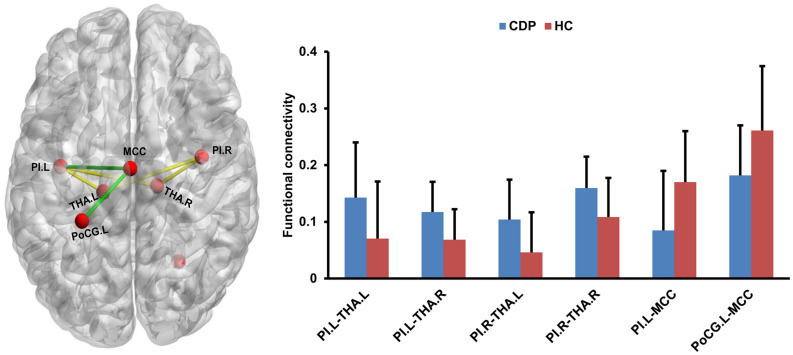
Changed resting-state functional connectivities within pain network. CDP patients exhibited increased functional connectivities between bilateral posterior insula (PI) and bilateral thalamus (THA.L and THA.R) compared to healthy controls. Moreover, decreased functional connectivities between the middle cingulate cortex (MCC) and left postcentral gyrus (PoCG.L), left PI were also found in CDP patients.

Seed to target functional connectivity analyses found a significant increase in functional connectivities between bilateral PIs and bilateral THA in CDP patients as compared to healthy controls (Figure 4). Significantly reduced functional connectivities between MCC and left PI, PoCG.L were also identified in CDP patients (Figure 4).

### Correlation Analyses

**FIGURE 5:**
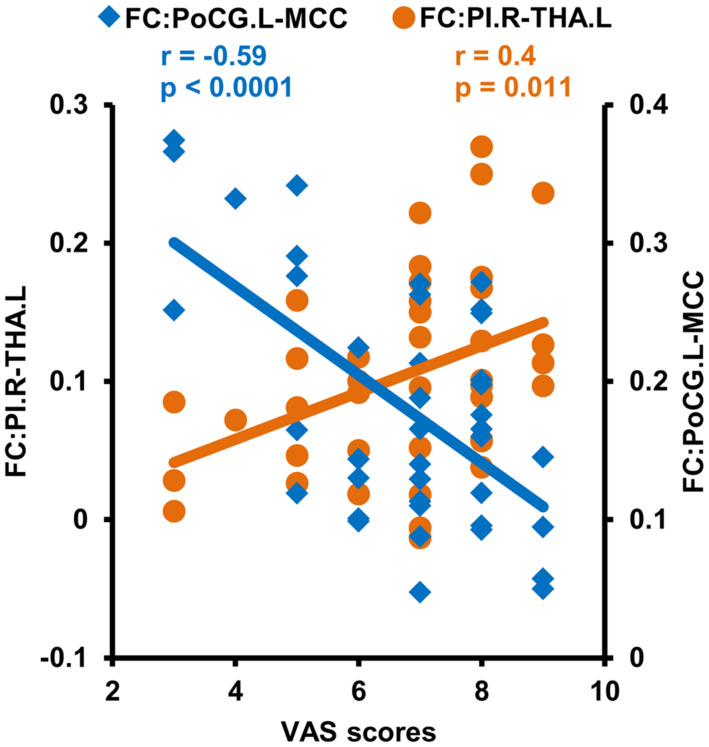
Correlation analyses between functional connectivities and clinical measures. Correlation analyses identified a significantly positive correlation between VAS scores and functional connectivities of the right posterior insula (PI.R) with left thalamus (THA.L), and negative correlations between VAS scores and the functional connectivities of the middle cingulate cortex (MCC) to left postcentral gyrus (PoCG.L) in CDP patients.

Correlation analyses identified a negative correlation between the functional connectivities of PoCG.L to MCC and VAS scores after multiple comparison corrections (*r* = −0.59, *p* = 0.0001). The positive correlation between the functional connectivities of THA.L to PI.R and VAS scores in CDP patients was found but not significant after multiple comparison correction (*r* = 0.4, *p* = 0.011) (Figure 5).

## Discussion

In this study, we identified different changing patterns within the pain network, including increased functional connections between bilateral PIs and bilateral THA and decreased functional connections between MCC and left PI, left PoCG in CDP patients. Moreover, the changed functional connections within the pain network were closely associated with pain intensity, i.e., VAS, scores. Our findings revealed changed patterns in the pain network of CDP patients, which may provide the neurophysiological basis and facilitate understanding of the neuropathology of CDP.

We found increased functional connectivities between bilateral posterior insula and thalamus in CDP patients. The posterior insula functionally connected with primary and secondary somatomotor cortices and medial thalamus is important for processing touch, pain, and thermal stimulation (Dosenbach et al., 2007; Thielscher and Pessoa, 2007; Deen et al., 2011; Kelly et al., 2012). The posterior insula is also a critical site for interoception and triggers the pain matrix network for subjective pain experience (Taylor et al., 2009; Isnard et al., 2011; Favilla et al., 2014). The thalamus is a relay for information exchanges between cortical and subcortical areas and plays a key role in the regulation of consciousness (Steriade and Llinas, 1988; Hwang et al., 2017; Lipiec and Wisniewska, 2019; Redinbaugh et al., 2020; Tang et al., 2020). The thalamus, which receives projections from multiple ascending pain pathways for modulating nociceptive information, is mainly involved in the sensory discriminative and affective motivational components of pain (Ab Aziz and Ahmad, 2006). A study using magnetic resonance imaging found that people with neuropathic pain showed reduced GABA in the thalamus (Henderson et al., 2013). The abnormal static and dynamic amplitude of low frequency fluctuations in CDP were also found in our previous study (Ma et al., 2020). All the evidence indicated that the abnormal functional activities or couplings of the insula and thalamus may be a neuromarker for CDP. Moreover, the functional connectivities between the posterior insula and thalamus were positively correlated with pain severity, suggesting that increased functional connectivities may be related to over sensitivity to pain stimuli in CDP patients.

The decreased functional connectivities between the middle cingulate cortex and left posterior insula, and left postcentral gyrus were found in CDP patients. Our finding was supported by previous studies which found changed structures and functions in these areas in cervical spondylosis and chronic knee osteoarthritis (Liao et al., 2018; Woodworth et al., 2019; Ma et al., 2020). The middle cingulate cortex has been reported in pain processing and is considered to be a key region in the pain matrix (Peyron et al., 2000; Favilla et al., 2014). The middle cingulate cortex is a part of the medial pain subsystem and is mainly involved in the effective and/or cognitive dimensions of pain processing (Frot et al., 2008). The postcentral gyrus is a part of the sensorimotor network involved in processing changing pain intensity and discrimination of the sensory components of pain perception (Kanda et al., 2000; Peyron et al., 2000). Thus, the decreased functional connectivity between middle cingulate cortex and postcentral gyrus, the posterior insula may be related to damaged cognitive and attention processing as well as disrupted pain modulating in CDP patients.

This study has some limitations. First, the selection of the sliding window length remains a topic of debate and the optimal length for obtaining the dynamics of brain activity is unclear. We selected 50 TR as the window length based on the criteria that the minimum length should be larger than 1/fmin, which was proposed in previous studies (Leonardi and Van De Ville, 2015; Li et al., 2019). The results of different sliding window lengths were similar to the main results of 50 TR, demonstrating that our findings of dALFF variability were relatively stable. Second, given the high comorbidity of anxiety and depression, excluding individuals with depressive disorder from future studies could decrease the generalizability of our findings. More comorbid samples are required to replicate and complement these findings. Third, seed based functional connectivity analysis of the posterior insula was used to define the pain network, and whether all the pain related brain areas were recruited needs further validation, which may bias the current findings. Finally, all the CDP patients were still under medication, which may affect the current findings.

## Conclusion

The current study revealed the changed intrinsic couplings within the pain network in CDP patients. We found increased functional couplings between the bilateral posterior insula and bilateral thalamus and decreased functional couplings between the middle cingulate cortex and postcentral gyrus, posterior insula in CDP patients. These increased functional connections may reflect over sensitivity to pain stimuli, whereas the decreased functional connections may be related to impaired pain modulating in cognition and emotion. Our findings provide initial evidence for the neural basis of CDP and could facilitate future understanding of the neuropathology of CDP.

## Data Availability Statement

The raw data supporting The conclusions of this article will be made available by the authors, without undue reservation.

## Ethics Statement

The studies involving human participants were reviewed and approved by The Affiliated Xi’an Central Hospital of Xi’an Jiaotong University. The patients/participants provided their written informed consent to participate in this study.

## Author Contributions

MM and SC designed this study. HZ, DX, XW, and RL analyzed the data and wrote the manuscript. HL, XYa, and XYi collected the data. All authors discussed and edited the manuscript.

## Conflict of Interest

The authors declare that the research was conducted in the absence of any commercial or financial relationships that could be construed as a potential conflict of interest.
